# Long-Term Outpatient Epidural Analgesia in Advanced Colorectal Adenocarcinoma: A Case Report

**DOI:** 10.7759/cureus.109883

**Published:** 2026-05-29

**Authors:** Ricardo Toipa Lopes, Noélia Carrillo-Alfonso, Cláudia Martins, Ana Lares

**Affiliations:** 1 Anesthesiology, Centro Hospitalar Universitário do Algarve, Faro, PRT

**Keywords:** advanced colorectal cancer, epidural analgesia, outpatient pain management, palliative care, refractory cancer pain

## Abstract

Pain is highly prevalent among patients with advanced cancer and often remains refractory to optimized systemic analgesia. When opioid therapy fails or causes intolerable side effects, interventional techniques may offer improved symptom control and quality of life. We report the case of a 73-year-old man with metastatic colorectal carcinoma who presented with lumbar plexus compression and severe refractory gluteal pain secondary to sacral tumor invasion and infiltration of the musculotendinous structures. Despite optimized systemic analgesia, the patient continued to experience pain ≥7/10, accompanied by a progressive decline in functional status. An epidural catheter was placed and connected to a patient-controlled epidural analgesia system delivering ropivacaine. This intervention enabled home-based management with scheduled follow-up. The patient had effective pain relief, allowing for the recovery of functional capacity. Dose adjustments were made as the disease progressed. The patient remained at home with sustained analgesic benefit until his death due to cancer progression. This case supports the role of outpatient epidural analgesia as an effective and safe palliative option for selected terminal cancer patients with uncontrolled pain, particularly when mobility and quality of life can be preserved.

## Introduction

Pain is one of the most common and distressing symptoms among patients with cancer, particularly in advanced stages of the disease, significantly affecting physical function and emotional well-being. The prevalence of cancer-related pain ranges from approximately 33% after curative treatment to up to 64% in patients with metastatic or terminal disease [1]. Cancer pain may result from tumor invasion, complications of malignancy, or treatment-related effects, and often involves nociceptive, neuropathic, or mixed mechanisms [1-3]. The World Health Organization analgesic ladder recommends a stepwise approach to pain management, progressing from non-opioid analgesics to weak opioids and ultimately strong opioids, with interventional techniques considered when pain remains inadequately controlled [1]. Despite optimized pharmacologic therapy, approximately 10% of patients experience pain that remains refractory to systemic treatment [3]. In such cases, interventional approaches including neuraxial analgesia may provide effective symptom control while minimizing systemic opioid-related adverse effects. This report describes the use of long-term outpatient epidural analgesia in a patient with advanced colorectal cancer and refractory gluteal pain secondary to lumbosacral plexus compression, highlighting its role in improving functional status and enabling home-based palliative care.

## Case presentation

A previously independent 73-year-old man (70 kg, 175 cm) with metastatic colorectal cancer was referred for a chronic pain appointment for evaluation of refractory pain. He was diagnosed with rectal adenocarcinoma with hepatic and pulmonary metastases. He was submitted to radiotherapy and chemotherapy and then to a two-time rectal resection. The tumor became unresectable, so he was shifted to palliative chemotherapy.

The patient developed a strong persistent left gluteal pain unresponsive to radiotherapy and escalating systemic analgesia. At the first consultation, Day 0, he reported constant left gluteal pain with average intensity 7/10 (range 5-10/10, Numeric Rating Scale (NRS)). The pain was relieved only in the prone position, which drastically impaired the patient's mobility. Current analgesics included gabapentin 300 mg 8/8 hours, naproxen 50 mg 8/8 hours, paracetamol 1,000 mg 12/12 hours, transdermal fentanyl 75 mcg/h, and sublingual fentanyl two rescue doses/day. Imaging showed a lesion in the precoccygeal space, with a diameter of 48 x 51 mm, with a secondary lesion in the sacrum, with a diameter of 67 x 76 mm, with an intraforaminal/subarachnoid component with partial obliteration of the intervertebral foramina on the left. This lesion was responsible for the current clinical situation, given the compression on the lumbosacral plexus on the same side. There was also an extensive extraosseous component in a posterior and lateral left location, with infiltration of the various musculotendinous structures on the same side.

An epidural catheter to manage the cancer-related pain was proposed and accepted. After 24 hours, Day 1, the patient was admitted for the procedure. Cefazoline 2 g was administered prophylactically. Under aseptic technique, an epidural catheter (Perifix®, B. Braun, Melsungen, Germany) was placed at L2-L3 (7.5 cm into the epidural space, 13 cm skin-to-epidural depth) in the caudal direction, confirmed by contrast fluoroscopy (Figures 1, 2). A test dose of 6 mL ropivacaine 2 mg/mL was administered, followed by 2 mL for insufficient analgesia relief. The catheter was subcutaneously tunneled for the right flank using the Tuohy needle. A “lock-it” sticker was applied and covered with a waterproof dressing. At the end of the procedure, the patient reported gluteal pain improvement (5/10 NRS).

**Figure 1:**
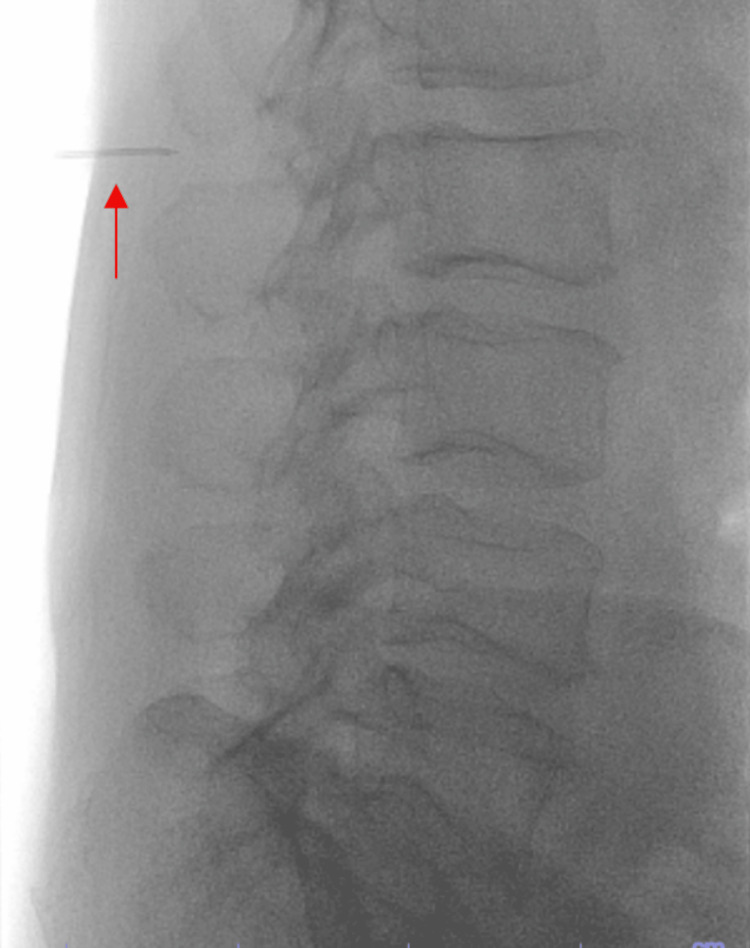
Insertion of the Tuohy needle (red arrow) at L2-L3 in the medial interlaminar approach.

**Figure 2:**
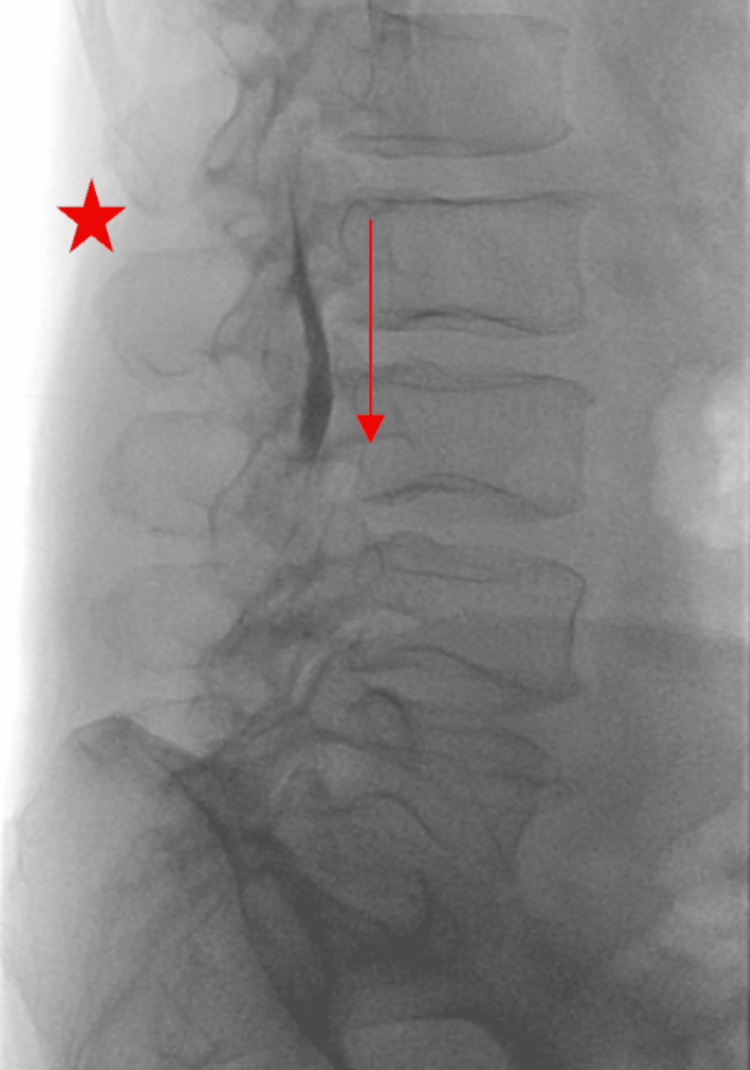
Contrast fluoroscopy showing the epidural location of the catheter (red star: catheter insertion at L2-L3; red arrow: catheter directed caudally).

A patient-controlled analgesia (PCA) infusion pump (CADD-Solis, ICU Medical, San Clemente, CA, USA) was programmed in a patient-controlled epidural analgesia (PCEA) mode, delivering an 8 mL bolus of ropivacaine 2 mg/mL with a lock-out of 20 minutes, with a maximum of 20 mL per hour with a 250 mL reservoir bag. The patient received education on catheter care, pump operation, and warning signs. The patient was discharged home with no motor or sensory block. Pain improvement was noted within 24 hours (2-3/10 NRS).

At follow-up on Day 8, the patient required an average of five boluses/day, with no motor or sensory impairment; dressings were changed, and a 250 mL reservoir bag was replaced; bolus volume was increased to 10 mL. On Day 10 and Day 15, pain control remained improved (2-3/10 NRS) with an average of four boluses/day with mild transient tingling post-bolus, without functional limitation. Dressings showed no inflammation, and the reservoir bag volume was changed to 500 mL. Collaboration was established with outpatient palliative care to perform dressing changes and catheter surveillance and provide medication. Telephone follow-ups occurred every two weeks. During this period, the patient regained some independence with the improved pain control, allowing prolonged sitting and to resume palliative chemotherapy, which had previously been suspended due to nausea.

On Day 30, as a result of increased pain (6/10 NRS), the bolus dose was increased to 12 mL. On Day 36, he reported further improvement (2-3/10 NRS) with 5-6 boluses/day and no adverse effects. The patient continued the nursing care in the palliative center without incidents.

On Day 66, the patient presented to the emergency department with left-leg weakness, constipation, and urinary retention. An epidural abscess was suspected, so an urgent computed tomography (CT) scan (Figures 3, 4) was performed, which showed sacral osteolysis with diffuse compression of the central and foraminal canal and consequent probable intrinsic neural and meningeal compression (sacral and coccygeal nerves). There was no evidence of signs of disc extrusion, synovial rupture, or unequivocally acute ligamentous rupture, namely, with neuronal mass effect or acute segmental instability. There was no evidence of intra- or peri-spinal volumetric or densitometric abnormalities compatible with pathological collections of traumatic origin and acute character. There were no signs of infection on the blood analyses. The case was discussed with the neurosurgery team, which excluded epidural abscess or catheter-related complications. Thus, progression of the underlying disease was assumed. The infusion protocol was adjusted to a 15 mL bolus of ropivacaine 1.5 mg/mL, with the reservoir volume increased to 1,000 mL. Reservoir bag and dressing changes were performed every 9-11 days in the outpatient palliative care setting. Despite disease progression (nausea, appetite and weight loss), pain control remained adequate (2-3/10 NRS).

**Figure 3:**
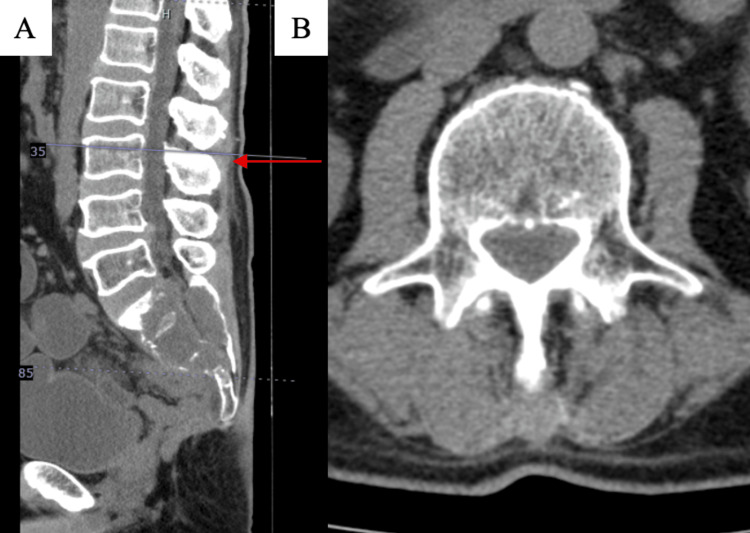
Computed tomography scan showing a (A) medial sagittal view of the lumbar spine and sacrum (straight line: superior vertebral body of L3 vertebrae) and an (B) axial view of the lumbar spine at the L3 level (red arrow) demonstrating the absence of signs of medullary compression.

**Figure 4:**
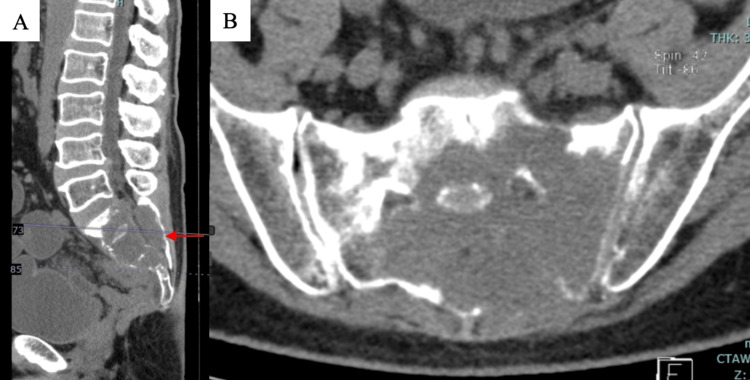
Computed tomography scan demonstrating a (A) medial sagittal view of the lumbar spine and sacrum level (straight line: superior vertebral body of S1 vertebrae) and an (B) axial view of the sacrum at the same level of cross-section (red arrow) showing local tumor invasion.

On Day 104, due to caregiver request, the delivery mode was changed to programmed intermittent epidural bolus (PIEB) of 20 mL every three hours with an optional 20 mL PCA bolus. The following day, no rescue boluses were needed. The patient continued home care under palliative follow-up. On Day 112, he died at home, with no reports of uncontrolled pain, requiring only as-needed morphine for the management of shortness of breath.

A detailed timeline of clinical events, analgesic interventions, and patient outcomes is presented in Table 1.

**Table 1:** Chronological sequence of clinical events based on follow-up data. ER: emergency department; NRS: Numeric Rating Scale; PCEA: patient-controlled epidural analgesia; PIEB: programmed intermittent epidural bolus

Timeline	Event	Intervention	Outcome
Day 0	Constant left gluteal pain 7/10 NRS (range 5-10/10).	First consultation. Transdermal fentanyl 75 mcg/h every 3 days. SOS sublingual fentanyl 100 mcg (2 per day). Oral analgesics: gabapentin 300 mg 8/8 h, naproxen 50 mg 8/8 h, paracetamol 1,000 mg 12/12 h.	Refractory pain despite optimized therapy. Epidural catheter proposed and accepted.
Day 1	Epidural catheter placement (medial interlaminar approach, L2-L3, catheter 7.5 cm epidural space, caudal direction).	PCEA initiated: ropivacaine 2 mg/mL, 8 mL bolus, 20 min lockout interval, max 20 mL/h, 250 mL reservoir. No changes to systemic medication.	Pain improvement. 5/10 NRS after procedure. 2-3/10 NRS within 24 hours.
Day 8	Mean 5 boluses/day.	PCEA bolus increased to 10 mL. New 250 mL reservoir installed.	Sustained pain control 2-3/10 NRS. Epidural regimen optimized.
Day 15	Mean 4 boluses/day.	Reservoir volume increased to 500 mL. Initiation of outpatient palliative care follow-up. Telephone reassessments every 2 weeks.	Stable pain control 2-3/10 NRS. Reduced bolus demand.
Day 30	Worsening pain 6/10 NRS.	PCEA bolus increased to 12 mL.	Pain improvement 2-3/10 NRS.
Day 36	Mean 5-6 boluses/day.	No changes.	Dose escalation required due to disease progression.
Day 66	ER admission: left lower limb weakness, constipation, and urinary retention.	PCEA adjusted: ropivacaine 1.5 mg/mL, bolus increase to 15 mL, 1,000 mL reservoir.	Disease progression. Neurological symptoms managed. Epidural regimen optimized.
Day 104	Mean 6-7 boluses/day.	Transitioned to PIEB (caregiver request): ropivacaine 1.5 mg/mL, 20 mL every 3 h, with 20 mL patient-controlled analgesic bolus.	Simplified administration. Maintained pain control 2-3/10 NRS.
Day 112	Death at home with no reports of uncontrolled pain.	Morphine for shortness of breath.	Comfortable end-of-life care achieved at home.

## Discussion

Poorly controlled pain is a major driver of healthcare services among cancer patients and is often worse in the home setting despite being the preferred place of care and death. Access to a multidisciplinary team improves pain management for patients living at home [3].

Although strong opioids are standard for advanced cancer pain, they may be insufficient for pain control at optimized doses or poorly tolerated [2,3]. Our patient had already been prescribed transdermal and sublingual fentanyl, which did not had any effect on pain control at the time of our consultation. Interventional approaches-including neurolysis and neuraxial infusions-can provide relief in selected patients [3]. The main concerns of neurolysis include neuritis, deafferentation pain, worsening existing symptoms, and motor weakness or bowel/bladder incontinence if motor/autonomic nerves are involved [1]. Neurolysis was avoided due to the risk of motor and autonomic impairment, given lumbar plexus involvement in this case. Intrathecal drug delivery may be through a single bolus using an external infusion pump or a fully implantable device. The choice of delivery method depends largely on the clinical situation and local nursing expertise and availability [1]. This approach was not pursued due to the high cost of the required equipment, its unavailability at our institution, and significant logistical constraints. The use of epidural catheters for the management of refractory cancer pain is not a novel concept and has been described for several decades in the pain medicine literature [4-7]. During the late 1980s and early 1990s, prolonged epidural analgesia became an established option for selected oncology patients with uncontrolled pain, particularly in specialized pain centers. Limitations associated with standard epidural catheters led to the development of silicone-based tunneled catheters, including the work pioneered by Stuart DuPen, aimed at improving durability and reducing infectious and mechanical complications [8]. The clinical relevance of these approaches was sufficiently recognized that they were incorporated into major reference textbooks of the era, including *Cancer Pain* (1993) by Richard Patt and *Interventional Pain Management* (1996) by Waldman and Winnie, both of which dedicated substantial discussion to the indications, technical considerations, benefits, and complications of long-term epidural analgesia in oncology patients. More recent studies have reinforced the described benefits in patients with intractable terminal cancer pain [9-12]. In patients with chronic cancer pain, epidural infusion resulted in sustained improvement over several weeks. Additionally, these patients experienced fewer systemic opioid-related side effects such as drowsiness and mental clouding, corresponding to a substantial reduction in oral opioid requirement [11,12]. More recent observational data in hospice patients with end-of-life refractory pain also demonstrated reduced opioid consumption alongside improved pain control following epidural analgesia, suggesting benefits in comfort, quality of life, and mitigation of opioid-associated adverse effects [9].

In this case, epidural analgesia was selected as the most appropriate option given the patient's preserved mobility, months-long life expectancy, and the need to maintain quality of life at home. The use of a patient-controlled system, available at the hospital, enabled the patient to self-administer medication via automated bolus dosing, ensuring safety through a lockout mechanism and limiting the maximum dose delivered within a closed system. This approach required only a single intervention, followed by scheduled evaluations, enabling continued home-based care.

Consistent with the literature on long-term outpatient epidural use, our main concerns were infection and catheter removal. Epidural and intrathecal catheter colonization rates range from 4.2% to 29%, although the incidence of central nervous system infection following neuraxial block remains rare, occurring in approximately 1-4.9 per 100,000 cases across various patient populations [13]. Retrospective studies report catheter infection rates of up to 22% in long-term epidural catheter use and accidental dislodgement rates ranging from 13% to 15% [13,14].

Infection is more likely in prolonged use and when tunnelling is not performed, and could range from superficial infections to epidural abscesses [10,11]. Therefore, patients and caregivers must be educated regarding wound care and early signs of infection or catheter-related complications [11]. The procedure was performed under aseptic conditions, and prophylactic antibiotic therapy was administered as recommended by the 2025 American Society of Regional Anesthesia and Pain Medicine Guidelines [13]. After a proper confirmation of the location by contrast fluoroscopy, the catheter was tunneled to reduce both the risk of infection and dislocation. The patient was instructed on dressing local infection and warning signs of neuraxial complications. He received regular follow-up by the palliative care nursing team, during which dressing changes and reservoir bag replacement were performed under aseptic conditions. The progressive increase in the reservoir bag volume avoided unnecessary manipulation of the system as larger boluses were needed.

The progressively increasing requirement for ropivacaine has raised an additional concern regarding the risk of local anesthetic systemic toxicity (LAST). Elderly patients are at increased risk of LAST due to reduced clearance of local anesthetics, resulting from age-related declines in metabolic organ perfusion and pharmacodynamic function [15]. Hypoalbuminemia may reduce the threshold for systemic toxicity by diminishing protein binding, thereby leading to an increased free fraction of local anesthetic in plasma [16]. Emerging evidence suggests that skeletal muscle functions as an important reservoir for local anesthetic storage. Therefore, reduced muscle mass limits this buffering capacity and increases circulating free drug levels [15,17]. In patients with advanced cancer receiving palliative care, approximately 32.4% present with sarcopenia associated with poorer nutritional status and reduced serum albumin levels [18]. The concurrence of those factors-advanced age, reduced muscle mass, and malnutrition-in our patient likely elevated the susceptibility to LAST. To mitigate that risk, a closed system with defined lockout intervals and maximum dose limits was employed. As the bolus volume was increased, the concentration of ropivacaine was reduced. To further minimize the daily total dose of local anesthetic and the frequency of bolus administration, an agent with a longer duration of action, such as levobupivacaine, could be chosen. Nevertheless, ropivacaine was chosen over levobupivacaine due to its lower systemic and cardiovascular toxicity, reduced motor blockade, and safer profile for continuous infusion, despite levobupivacaine offering slightly greater potency but with a marginally higher risk and motor effects. In oncology patients, particularly in the context of refractory pain or palliative care, the decision to employ local anesthetic-based techniques should be guided by an individualized assessment of the balance between clinically meaningful benefits and potential risks. These techniques provide significant benefits, such as effective control of refractory pain, avoidance of opioid dose escalation, preservation of functional capacity, the possibility of maintaining oncological treatments, and improvement in quality of life, despite the inherent risks. Although the occurrence of LAST remains rare, a high index of suspicion should always be maintained, and the risk can be mitigated through weight- and condition-adjusted dosing, the use of anesthetics with favorable safety profiles, and appropriate clinical monitoring [19].

An adjuvant could also be administered alongside the local anesthetic. Epidural morphine provides segmental analgesia through direct action on the spinal cord, which is particularly beneficial for nociceptive and refractory cancer pain. When combined with epidural local anesthetics, this approach produces an analgesic synergy, permitting the use of lower doses of each agent and thereby minimizing systemic adverse effects [10,11]. However, the patient chose not to pursue this approach, and pain control had been adequately maintained with fentanyl.

The implementation of an outpatient epidural presented significant challenges in this low-income setting. Although a PCA system was available, logistical barriers to follow-up care and regular catheter/dressing maintenance, limited availability of staff trained in advanced pain management techniques, and the absence of standardized protocols posed substantial obstacles to safe and effective management. To address these challenges, collaboration with the palliative care team was established to ensure ongoing patient follow-up and medication supply, provide education to healthcare professionals on neuraxial care, and identify potential complications early. Despite these limitations, structured coordination and adherence allowed for safe and effective use of the epidural.

As a single case report, the findings cannot be generalized to the broader population of patients with refractory cancer pain. Improvements in pain control, functional status, and quality of life were based on clinical observation and patient-reported outcomes rather than formally validated instruments, which limits the objectivity of the reported outcomes. Additionally, the absence of a standardized protocol for long-term outpatient epidural management at our institution means that the approach described was largely individualized, and its reproducibility in other settings remains uncertain. In the end, outpatient epidural analgesia offered sustained relief, preserved autonomy, and prevented opioid escalation, supporting its role in palliative care where functional status and home stay are priorities.

## Conclusions

This case illustrates that long-term outpatient epidural analgesia may be feasible even in low-resource settings without specialized systems or the infrastructure typically available in major tertiary centers. With appropriate interdisciplinary support, caregiver education, and careful surveillance for complications, this approach may be considered as an option outside specialized centers, offering effective pain control while preserving comfort, functional status, and home-based end-of-life care for selected terminal cancer patients with refractory pain or intolerable opioid-related side effects.
